# Neural Response to Food Cues in Avoidant/Restrictive Food Intake Disorder

**DOI:** 10.1001/jamanetworkopen.2024.60101

**Published:** 2025-02-18

**Authors:** Jennifer J. Thomas, Laura Holsen, Avery L. Van De Water, Kendra R. Becker, Lauren Breithaupt, Helen Burton-Murray, Elisa Asanza, Julia Gydus, Lilian P. Palmer, Casey M. Stern, Melissa Freizinger, Lydia A. Shrier, Elana M. Bern, Thilo Deckersbach, Madhusmita Misra, Kamryn T. Eddy, Nadia Micali, Elizabeth Lawson

**Affiliations:** 1 Eating Disorders Clinical and Research Program, Massachusetts General Hospital, Boston; 2 Mass General Brigham Multidisciplinary Eating Disorders Research Collaborative, Boston, Massachusetts; 3 Harvard Medical School, Boston, Massachusetts; 4 Division of Women’s Health, Department of Medicine, and Department of Psychiatry, Brigham and Women’s Hospital, Boston, Massachusetts; 5 Neuroendocrine Unit, Massachusetts General Hospital, Boston; 6 Center for Neurointestinal Health, Division of Gastroenterology, Massachusetts General Hospital, Boston; 7 Department of Psychiatry and Behavioral Sciences, Boston Children’s Hospital, Boston, Massachusetts; 8 Division of Adolescent/Young Adult Medicine, Boston Children’s Hospital, Boston, Massachusetts; 9 Division of Gastroenterology, Hepatology and Nutrition, Boston Children’s Hospital, Boston, Massachusetts; 10 Division of Neurotherapeutics, Massachusetts General Hospital, Charlestown; 11 University of Applied Sciences, DIPLOMA Hochschule, Bad Sooden-Allendorf, Germany; 12 Department of Pediatrics, University of Virginia, Charlottesville; 13 Eating Disorders Research Unit, Mental Health Center Ballerup, Mental Health Services of the Capital Region of Denmark, Ballerup, Denmark; 14 Great Ormond Street Institute of Child Health, University College London, London, United Kingdom

## Abstract

**Importance:**

The neurobiology of avoidant/restrictive food intake disorder (ARFID) is poorly understood.

**Objective:**

To evaluate whether individuals with ARFID exhibit disruptions in fear, appetite, and disgust brain regions compared with healthy control (HC) participants when shown images of food and objects.

**Design, Setting, and Participants:**

In this case-control study conducted from July 2016 to January 2021, children, adolescents, and young adults completed structured interviews and a validated functional magnetic resonance imaging (fMRI) food cue paradigm. The study was conducted at a single academic medical center. Data analysis was conducted from April 2023 to August 2024.

**Exposures:**

Presence vs absence of ARFID and its phenotypes (ARFID-fear, ARFID–lack of interest in eating, ARFID–sensory sensitivity); pictures of food vs objects during fMRI food cue paradigm.

**Main Outcomes and Measures:**

Blood oxygenation level–dependent activation in regions of interest (ROIs; amygdala, hypothalamus, insula, anterior cingulate cortex [ACC]) and the whole brain.

**Results:**

Participants were 110 children, adolescents, and young adults with full or subthreshold ARFID (75 participants; mean [SD] age, 16.2 [3.8] years; 41 [55%] female) and age-matched HC participants (35 participants; mean [SD] age, 17.3 [4.0] years; 27 [69%] female) recruited for studies of the neurobiology of ARFID and restrictive eating disorders. Participants with ARFID demonstrated greater activation than HC participants of the ACC (mean difference, 0.48 [95% CI, 0.19 to 0.77]; *P* = .009), sensory association cortex (mean difference on left side, 0.54 [95% CI, 0.29 to 0.79]; *P* = .005; right side, 0.52 [95% CI, 0.28 to 0.76]; *P* = .02), and supplementary motor cortex (mean difference, 0.81 [95% CI, 0.47 to 1.15]; *P* = .04). The ARFID-fear group showed greater amygdala activation vs HC (mean difference, 0.49 [95% CI, 0.16 to 0.82]; *P* = .04), and greater lack of interest was associated with lower hypothalamus activation in the ARFID–lack of interest group (*r* = −0.38 [95% CI, −0.69 to −0.11]; *P* = .03). The ARFID–sensory sensitivity group did not show greater insula activation vs HC but showed greater activation of the ACC (mean difference, 0.48 [95% CI, 0.22 to 0.74]; *P* = .005) and somatosensory cortex (mean difference on left side, 0.60 [95% CI, 0.33-0.87]; *P* = .001; right side, 0.54 [95% CI, 0.29 to 0.80]; *P* = .03).

**Conclusions and Relevance:**

Results indicate generalized hyperactivation of ACC, sensory association cortex, and supplementary motor cortex in response to visual food stimuli in children, adolescents, and young adults with ARFID, suggesting a novel neurobiological circuit associated with this disorder. Activation appears consistent with ARFID phenotypic rationales for food avoidance, with hyperactivation of fear regions in ARFID-fear and hypoactivation of appetite regions with increasing ARFID–lack of interest severity.

## Key Points

**Question:**

Does neural activation differ among individuals with avoidant/restrictive food intake disorder (ARFID) and healthy control (HC) participants and across ARFID phenotypes?

**Findings:**

In this case-control study of 110 children, adolescents, and young adults (75 with ARFID and 35 HC participants), those with ARFID demonstrated greater activation of the anterior cingulate cortex (ACC), sensory association cortex, and supplementary motor cortex; those with the ARFID-fear phenotype showed greater amygdala activation; greater lack of interest in those with the ARFID–lack of interest phenotypes was associated with lower hypothalamus activation; and those with the ARFID–sensory sensitivity phenotype exhibited greater activation of the ACC, somatosensory cortex, and supplementary motor cortex.

**Meaning:**

In this study, relative to healthy individuals, individuals with ARFID exhibited hyperactivation within a novel neurobiological circuit associated with aversive conditioning, attention, and multisensory processing.

## Teaser

This case-control study compares functional magnetic resonance imaging results from a food cue paradigm between youths with avoidant/restrictive food intake disorder (ARFID) and age-matched healthy control participants.

## Introduction

Avoidant/restrictive food intake disorder (ARFID) is a serious psychiatric condition that affects 0.3% to 15.5% of children^1^ and 0.3% to 4%^2,3^ of adults. ARFID leads to severe health consequences (weight loss or faltering growth, nutritional deficiencies, supplemental feeding dependence, and/or psychosocial impairment)^4^ and follows a persistent course.^5^ Characterizing neural activation in ARFID is essential for developing treatments that target disease mechanisms.

Informed by the Research Domain Criteria (RDoC),^6^ our team has proposed a 3-dimensional neurobiological model of ARFID in which the 3 *DSM-5* ARFID phenotypes—fear of aversive consequences (ARFID-fear), lack of interest in eating (ARFID–lack of interest), and sensory sensitivity (ARFID–sensory sensitivity)—occur along a continuum of severity and may co-occur within the same individual.^7^ The 3 phenotypes have been replicated by independent research teams,^8,9,10,11^ but their underlying neurobiology is poorly understood. Our model posits that ARFID is characterized by disruptions in fear, appetite, and disgust processing, and that disruptions differ by phenotype.

Specifically, individuals with the ARFID-fear presentation describe feeling terrified of food after traumatic experiences (eg, choking, vomiting).^4,12^ Activation of the brain’s defensive motivational system promotes behaviors that protect organisms from danger.^13^ However, overactivity in neural fear circuitry could lead to prolonged avoidance of conditioned stimuli that are no longer harmful. The amygdala is the hub of the subcortical fear circuit,^14,15^ and amygdala hyperactivation to fear stimuli has been reported for anxiety and stress-related disorders including specific phobia^16^ and posttraumatic stress disorder.^17^ Therefore, hyperactivation of fear regions—particularly the amygdala—may serve as a maintaining mechanism of ARFID-fear.

Individuals with ARFID–lack of interest describe early satiation and excessive fullness.^4,12^ RDoC arousal/regulatory systems are responsible for homeostatic regulation of appetite and energy balance. Homeostatic food intake is governed by hypothalamic nuclei, including the arcuate nucleus, paraventricular nucleus, and lateral hypothalamic area.^18^ In healthy individuals, satiation is associated with reduced hypothalamic activity, and individuals with anorexia nervosa (AN) show attenuated hypothalamic response after eating.^19^ Thus, hypothalamus hypoactivation in response to food stimuli may maintain ARFID–lack of interest.

Lastly, individuals with ARFID–sensory sensitivity limit food variety, describing many foods as disgusting. The anterior insula is involved in disgust processing^20^ and is reliably activated in healthy individuals in response to disgusting (eg, rotting, insect-infested) food.^21,22^ If disgust is also relevant to ARFID–sensory sensitivity, the insula may show inappropriate hyperactivation to images of food in general. The anterior cingulate cortex (ACC), which has anatomical connections with the insula, plays a role in attention and conflict monitoring. This region has been shown to mediate food-related decision-making,^23,24^ and enhanced activation of the ACC to food cues was noted among individuals prone to disgust^25^ and with dysphagia.^26^ Coupled with sensitivity in the insula to food cues, individuals with ARFID–sensory sensitivity may exhibit dysfunction in the ACC related to conflicting internal and external cues, particularly during states of hunger, as they must decide to consume or not consume foods that meet metabolic need but are considered disgusting.

In summary, no published data of which we are aware have investigated ARFID’s neural mechanisms. One small prior study^27^ compared fMRI response to food cues across the weight spectrum in full and subthreshold ARFID but did not compare ARFID with health control (HC) participants. In the current study, we used a validated visual food cue paradigm^28,29^ in which children and adolescents (either with full or subthreshold ARFID or HC participants) viewed pictures of food vs objects. First, we hypothesized that, compared with HC participants, individuals with ARFID would show hyperactivation of the amygdala, insula, and ACC as well as hypoactivation of the hypothalamus. Second, we hypothesized that, compared with HC participants, the ARFID-fear group would show greater activation of the amygdala (a key fear region of interest [ROI]) and that this hyperactivation would be positively correlated with severity of fear of aversive consequences. Third, we hypothesized that, compared with HC participants, the ARFID–lack of interest group would show hypoactivation in the hypothalamus (a key appetite ROI), and hypothalamus activation would be negatively correlated with severity of lack of interest. Lastly, we predicted that the ARFID–sensory sensitivity group would show hyperactivation of the insula and ACC (key disgust ROIs) and that greater insula hyperactivation would be positively correlated with sensory sensitivity severity.

## Methods

We followed the Strengthening the Reporting of Observational Studies in Epidemiology (STROBE) guidelines for reporting the methodology of this case-control study.^30^ The Mass General Brigham Human Research Committee approved the study. Adults (aged ≥18 years) provided written informed consent; children (aged ≤17 years) provided assent, and their parents provided consent.

### Participants

Participants with ARFID were drawn from a study of the neurobiology of ARFID (study 1). HC participants were drawn from the same study and a related study of the neurobiology of restrictive eating disorders (study 2). We matched participants on age by recruiting similar proportions of individuals with ARFID and HC participants across 3 age categories: preadolescence (ages 10-12 years), adolescence (ages 13-17 years), and young adult (ages 18-23 years). We recruited from July 2016 to January 2021 via online advertisements, pediatric practices, and eating disorder clinics. We based our sample size on a power analysis indicating a greater than 80% power to detect between-group differences of medium effect size (*d* = 0.50) at a significance level of .05 (1-sided) between ARFID and HC using an ROI approach.

Participants with full or subthreshold ARFID were included if they met criteria for ARFID on the Eating Disorder Assessment (EDA-5)^31^ for *Diagnostic and Statistical Manual of Mental Disorders* (Fifth Edition) (*DSM-5*) or endorsed ARFID symptoms on the Kiddie Schedule for Affective Disorders and Schizophrenia (KSADS).^32^ ARFID diagnoses were confirmed at the main study visit via the Pica, ARFID, and Rumination Disorder Interview (PARDI; in study 1)^33^ or Eating Disorder Examination (EDE)^34^ plus Longitudinal Interval Follow-up Evaluation (LIFE-EAT-3) modified to include ARFID symptoms^5,35^ (in study 2). HC participants were included if they did not endorse eating-disorder psychopathology on the EDA-5, KSADS, and either PARDI or EDE. All HC participants had a body mass index between the 10th and 90th percentiles, regular menstrual cycles, no history of delayed pubertal onset, less than 10 h/wk of exercise, and no lifetime history of any psychiatric disorder. Exclusion criteria for both ARFID and HC groups included current pregnancy or breastfeeding, lifetime psychosis, current substance use disorder, medical comorbidities that could affect study findings (eg, gastrointestinal tract surgery, hematocrit <30%, diabetes), and magnetic resonance imaging (MRI) contraindications.

### Procedure

After a phone screen, potentially eligible participants attended a screening visit at the Massachusetts General Hospital Clinical Research Center, where they completed a history and physical examination (height, weight) with a physician or nurse practitioner, pregnancy test, safety laboratory tests (hematocrit), KSADS, and Annette Hand Preference Questionnaire.^36^ Participants self-reported their race and ethnicity according to National Institute of Health guidelines. We collapsed American Indian or Alaska Native individuals and Native Hawaiian or Other Pacific Isalnder individuals in an other race group due to small sample size. After an overnight fast, eligible participants presented for the morning study visit at the Martinos Center for Biomedical Imaging, where they completed the functional MRI (fMRI) food cue paradigm (in a fasted state), and an eating-disorder interview (PARDI in study 1; or EDE in study 2). The main study visit included other procedures we have reported elsewhere (eg, blood draws at fasting and after a standardized test meal).^37,38,39^

### Measures

#### EDA-5

The EDA-5^31^ is an interview that confers eating disorder diagnoses. We used the EDA-5 at the study 1 screening visit to identify possible ARFID and to rule out other eating disorders.

#### KSADS–Present and Lifetime Version

The KSADS–Present and Lifetime Version (KSADS-PL) is an interview that assesses current and lifetime psychiatric diagnoses in youth.^32^ We used the KSADS-PL to assess inclusion and exclusion criteria.

#### PARDI

The PARDI interview produces ARFID diagnoses, and measures overall ARFID severity and severity of each ARFID phenotype.^33^ In prior studies, internal consistency for phenotype ratings has ranged from 0.72 to 1.0, and interrater reliability of the ARFID diagnosis has been excellent (100% agreement; κ = 1.0).^40,41^ We classified participants who restricted their intake by volume and/or variety, but did not meet criterion A to the severity required by the PARDI, as subthreshold ARFID. For example, participants who ate no fruits or vegetables, but endorsed mild (rather than marked) psychosocial impairment, were diagnosed with subthreshold ARFID. PARDI scores range from 0 (no symptoms) to 6 (extreme severity). The PARDI can also categorize individuals into ARFID phenotype groups based on recommendations from a receiver operating curve analysis (≥0.625 for ARFID–lack of interest; ≥1.125 for ARFID–sensory sensitivity).^42^ However, Cooper-Vince et al^42^ cautioned against using their cut point for ARFID-fear due to low sensitivity, so we defined the ARFID-fear phenotype as scoring 0.5 of higher, approximating the natural break in the distribution.

#### EDE

The EDE is a structured clinical interview for the specific psychopathology of eating disorders.^34^ We used the EDE to confirm HC vs ARFID status in study 2.

### fMRI Paradigm

Participants completed a validated visual food cue paradigm^19,28,29,43^ during blood oxygenation level–dependent (BOLD) data acquisition. This paradigm featured 100 high-calorie food images, 100 low-calorie food images, 100 nonfood images (household objects), and 100 fixation stimuli (blurred images) distributed across five 4-minute runs and presented in a block design. Each run consisted of 16 blocks, with 5 images per block. Within a block, images appeared for 3 seconds each, with block order counterbalanced within and across runs. Participants confirmed that they had seen the pictures by pressing a button when each picture changed. The primary contrast of interest was activation during food images (high- and low-calorie images, given that individuals with ARFID avoid both low- and high-calorie foods) vs nonfood images.^12^

### Acquisition Parameters

MRI data were acquired on a 3T Skyra scanner (Siemens) equipped with a 12-channel head coil. We constrained head movements with foam cushions. Whole-brain functional imaging data were acquired using a gradient echo planar imaging pulse sequence (33 contiguous oblique-axial slices; 4.0-mm thick; repetition time [TR]/time to echo [TE], 2000/30 milliseconds; flip angle, 85°; field of view, 216 × 216 mm). A sagittal T1-weighted 3D MPRAGE sequence (128 sagittal slices; 1.0-mm thick; TR/TE, 2530/3.43 milliseconds; flip angle, 7°; field of view, 256 × 256 mm; effective slice thickness, 1.33 mm) was used for coregistration between structural and functional data.

### Statistical Analysis

fMRI data were analyzed using SPM12.^44^ We calculated descriptive statistics and demographic comparisons using R version 4.3.1 (R Project for Statistical Computing).^45^ Functional data were unwarped with phase correction provided from the field map, slice-time corrected with reference to the midpoint slice, and realigned to the first volume. Structural T1-weighted images were segmented into different tissue classes (gray matter, white matter, cerebrospinal fluid, bone, soft tissues, and air), bias corrected for intensity inhomogeneities, and spatially normalized. Bias-corrected T1-weighted images were then skull stripped using SPM Image Calculator, entered as a reference image to coregister the mean realigned functional image of each participant, and normalized to the Montreal Neurological Institute (MNI) 152 brain template. Finally, functional data were resampled to 3 mm isotropic and smoothed with a 6-mm Gaussian kernel.

Following preprocessing, functional data were subjected to first-level modeling. Within the block design, each stimulus type was modeled using a boxcar function convolved with a canonical hemodynamic response function. Outliers in global mean image time series (threshold, 3.5 SDs) and movement (threshold, 0.8 mm, scan-to-scan movement) were detected using Artifact Detection Tools (ART)^46^ and entered as nuisance regressors in the single participant-level general linear model. First-level analyses were conducted on our contrast of interest (food vs nonfood images) using linear contrasts and SPM t-maps, which were then submitted to second-level random-effects group analysis.

In second-level analyses, we tested whether the ARFID group exhibited differential activation relative to the HC group using a 2-sample *t* test (1-sided). Results were interrogated first with a focus at the ROI level (applying a small-volume correction [SVC] threshold peak-level family-wise error [FWE] corrected *P* < .05) within the amygdala, hypothalamus, insula, and ACC, and then at the whole-brain level (initial voxelwise cluster-forming threshold, *P* = .001; cluster-level threshold, FWE-corrected *P* < .05). A priori ROIs were defined anatomically for amygdala, insula, and ACC using the Automated Anatomical Labeling (AAL3) atlas,^47^ and for the hypothalamus using a mask from the Center for Morphological Analysis at the Martinos Center.

Next, we examined hypotheses for each ARFID phenotype. First, a 2-sample *t* test (1-sided) compared individuals with each ARFID phenotype with the HC group on activation to food vs nonfood images, with ROI analyses focused on a priori ROIs for each phenotype (ie, ARFID-fear vs HC; ARFID–lack of interest vs HC; ARFID–sensory sensitivity vs HC). Second, a regression model (significance level *P* < .05) within each ARFID phenotype examined the linear association between symptom severity (operationalized as scores on the relevant PARDI subscale) and activation to food vs nonfood images. For regression models, PARDI subscale scores for the lack of interest and sensory sensitivity subscales were transformed due to nonnormal distributions (PARDI lack of interest, square root transformation; PARDI sensory sensitivity, natural log transformation).

Participants with ARFID could be included in more than 1 phenotype group, given evidence that ARFID phenotypes often overlap.^7,9,10,11^ We conducted models at the ROI level (SVC thresholds: FWE-corrected *P* < .05) to test our hypotheses within ROIs specified for each phenotype (ARFID-fear, amygdala; ARFID–lack of interest, hypothalamus; ARFID–sensory sensitivity, insula and ACC) and then at the whole-brain level (initial voxelwise cluster-forming threshold, *P* = .001; cluster-level threshold, FWE-corrected *P* < .05).

## Results

One hundred and ten children, adolescents, and young adults (aged 10-23 years; 65 [59%] female) with full or subthreshold ARFID (75 participants; mean [SD] age, 16.2 [2.8] years; 41 [55%] female) and age-matched HC participants (35 participants; mean [SD] age, 17.3 [4.0] years, 24 [69%] female) participated. Table 1 shows demographic and clinical characteristics, and eFigure 1 in Supplement 1 depicts participant flow.

**Table 1.** Demographic and Clinical Characteristics in Participants With ARFID and HC Participants

Characteristic	No. (%)		Test statistic	*P* value
	ARFID (n = 75)	HC (n = 35)		
Sex				
Male	34 (45)	11 (31)	χ^2^ = 1.38	.24
Female	41 (55)	24 (69)		
Age, mean (SD), y	16.2 (3.8)	17.3 (4.0)	*t* = −1.44	.15
Race^a^				
Asian	0	4 (12)	χ^2^ = 2.43	.12
Black/African American	1 (1)	1 (3)		
White	69 (92)	27 (79)		
Other	0	0		
More than 1 race	5 (7)	2 (6)		
Ethnicity^a^				
Hispanic	7 (9)	4 (12)	χ^2^ = .00	.96
Non-Hispanic	68 (91)	30 (88)		
BMI percentile, mean (SD)	39.2 (34.9)	51.7 (23.2)	*t* = −1.95	0.054
Handedness^b^				
Right	64 (85)	30 (86)	χ^2^ = 2.20	.33
Left	10 (13)	4 (11)		
Either	0	1 (3)		
PARDI scores, mean (SD)^c^				
Fear of aversive consequences	0.48 (0.87)	0	*t* = 6.25	<.001
Lack of interest in eating of food	2.10 (1.64)	0.14 (0.27)	*t* = 6.10	<.001
Sensory sensitivity	1.62 (1.30)	0.20 (0.10)	*t* = 6.25	<.001
ARFID presentation^d^^,^^e^				
Fear of aversive consequences	20 (27)	0	NA	NA
Lack of interest in eating of food	48 (64)	0		
Sensory sensitivity	58 (73)	0		
Current KSADS-PL diagnoses^d^				
Depressive and bipolar-related disorders	10 (13)	0	NA	NA
Anxiety, obsessive-compulsive, and trauma-related disorders	33 (44)	0		
Neurodevelopmental, disruptive, and conduct disorders	15 (20)	0		
Psychiatric pharmacotherapy^d^				
Antidepressants or anxiolytics	29 (39)	0	NA	NA
Antipsychotics	7 (9)	0		
Psychostimulants	13 (17)	0		

Abbreviations: ARFID, avoidant/restrictive food intake disorder; BMI, body mass index; HC, healthy control; KSADS-PL, Kiddie Schedule for Affective Disorders and Schizophrenia–Present and Lifetime Version; NA, not applicable; PARDI, Pica, ARFID, and Rumination Disorder Interview.

^a^ One individual with the healthy control group chose not to disclose their race and ethnicity. Due to small cell sizes (<5) for several race categories, groups were combined into White compared with all other race and ethnicity groups for statistical comparison between ARFID and HC.

^b^ Data for 74 participants with ARFID available. Handedness was measured with the Annette Handedness Questionnaire.

^c^ Data for 73 participants with ARFID and 26 HC participants..

^d^ ARFID and HC groups were not compared statistically on psychopathology variables because between-group differences were expected due to the case-control design.

^e^ Percentages add up to more than 100% because profiles can overlap.

### ARFID vs HC

**Figure 1.**
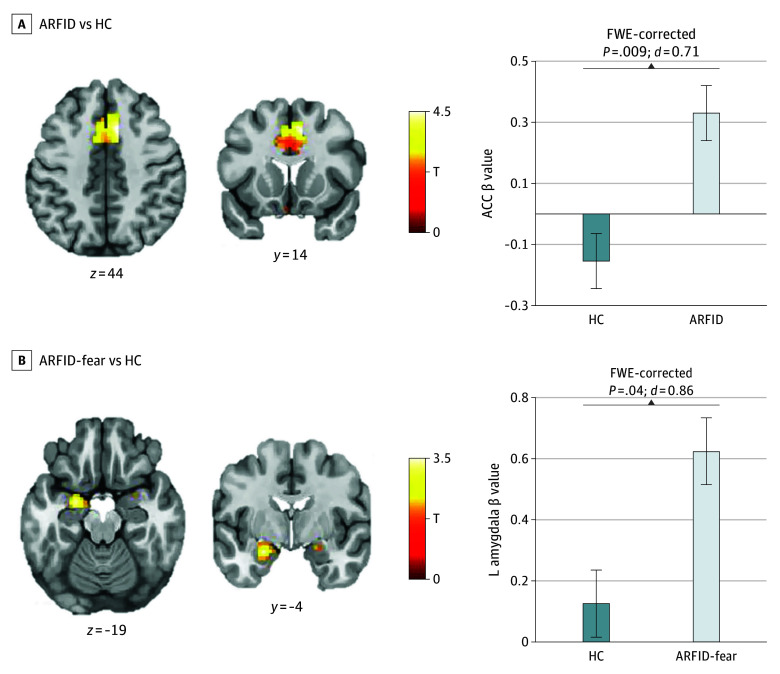
Blood Oxygenation Level–Dependent (BOLD) Activation to Food Cues in Participants With Avoidant/Restrictive Food Intake Disorder (ARFID) vs Healthy Control (HC) Participants and Those With the ARFID-Fear Phenotype vs HC Participants (A Priori Region-of-Interest [ROI] Analyses) A, Results showing significant differences between groups (ARFID and HC) in BOLD activation to foods vs objects in a priori ROIs. BOLD activation differed between groups (34 HC participants; 75 participants with ARFID) in the anterior cingulate cortex (ACC). B, Results showing significant differences between group (ARFID-fear and HC) in BOLD activation to foods vs objects in a priori ROIs. BOLD activation differed between groups (34 HC participants; 20 participants with ARFID-fear phenotype) in the amygdala. For both panels (A, B), the T scale and *P* value reflect the group difference from the independent samples *t* test. Statistical thresholding reflects small volume correction within an anatomically defined bilateral ROI at family-wise error (FWE)–corrected *P* < .05. Statistical maps for BOLD activation are overlaid on a normalized canonical image (Montreal Neurological Institute ICBM 152 nonlinear asymmetric T1 template) with SPM color map corresponding to relative T value. Coordinates (y and z) are presented in MNI space, with y corresponding to the coronal plane and z to the axial plane. The bar graph depicts mean β values within each cluster for each group, with whiskers indicating the SEM.

As hypothesized, the ARFID group demonstrated greater activation of the ACC, relative to the HC group, in response to food (vs nonfood) images (mean difference, 0.48 [95% CI, 0.19-0.77]; FWE-corrected *P* = .009 at ROI level) (Table 2, Figure 1A) with a medium-to-large effect size (*d* = 0.71). Contrary to hypotheses, activation in the remaining ROIs (amygdala, hypothalamus, insula) in response to food (vs nonfood) images did not differ between ARFID and HC groups. At the whole-brain level, there was hyperactivation in the ARFID group (vs HC) in the ACC (mean difference, 0.48 [95% CI, 0.29-0.69]; FWE-corrected *P* < .001), bilateral sensory association cortex (left mean difference, 0.54 [95% CI, 0.29-0.79]; FWE-corrected *P* = .005; right mean difference, 0.52 [95% CI, 0.28-0.76]; FWE-corrected *P* = .02), and supplementary motor cortex (mean difference, 0.81 [95% CI, 0.47-1.15]; FWE-corrected *P* = .04 (Table 3; eFigure 2 in Supplement 1).

**Table 2.** Blood Oxygenation Level–Dependent Activation to Food Cues in ARFID vs HC Participants (A Priori Region of Interest Analysis)

Comparison	R/L^b^	Peak *t* value	*k* ^c^	*P* value^d^	Coordinates^a^			*d*
					x	y	z	
HC vs ARFID^e^								
Amygdala	NA	NA	NA	NA	NA	NA	NA	NA
Hypothalamus	NA	NA	NA	NA	NA	NA	NA	NA
Insula	NA	NA	NA	NA	NA	NA	NA	NA
ACC	R	4.44	560	.009	9	14	44	0.71
HC vs ARFID-fear group^f^								
Amygdala	L	3.41	48	.04	−24	−4	−19	0.86
HC vs ARFID–lack of interest group^g^								
Hypothalamus	NA	NA	NA	NA	NA	NA	NA	NA
HC vs ARFID–sensory sensitivity group^h^								
Insula	NA	NA	NA	NA	NA	NA	NA	NA
ACC	R	4.70	582	.005	9	8	44	0.80

Abbreviations: ACC, anterior cingulate cortex; ARFID, avoidant/restrictive food intake disorder; HC, healthy control; L, left; NA, no significant cluster; R, right.

^a^ Coordinates are presented in Montreal Neurological Institute space.

^b^ R/L denotes hemisphere in which peak voxel within each cluster was localized.

^c^ Cluster size (contiguous voxels).

^d^ Statistical significance was assessed at *P* < .05, family-wise error–corrected, using small-volume correction.

^e^ Data for 35 HC participants and 75 participants with ARFID. No significant clusters for amygdala, hypothalamus, or insula.

^f^ Data for 35 HC participants and 20 participants with ARFID-fear phenotype.

^g^ Data for 35 HC participants and 48 participants with ARFID–lack of interest phenotype. No significant clusters for hypothalamus.

^h^ Data for 35 HC participants and 58 participants with ARFID–sensory sensitivity phenotype. No significant clusters for insula.

**Table 3.** Blood Oxygenation Level–Dependent Activation to Food Cues in ARFID vs HC Participants (Whole-Brain Analysis)

Comparison	R/L^b^	Peak *t* value	k^c^	*P* value^d^	Coordinates^a^			*d*
					x	y	z	
HC vs ARFID^e^								
ACC	R	4.44	189	<.001	9	14	44	0.99
Sensory association cortex/BA 5	L	4.55	107	.005	−9	−46	62	0.82
	R	4.21	76	.02	15	−46	62	0.85
Supplementary motor cortex/BA 6	R	3.8	66	.04	6	−1	68	1.00
HC vs ARFID-fear group^f^								
No significant clusters	NA	NA	NA	NA	NA	NA	NA	NA
HC vs ARFID–lack of interest group^g^								
ACC	R	4.53	156	.001	9	14	44	1.21
Sensory association cortex/BA 5	L	4.45	102	.006	−12	−46	62	0.92
	R	4.24	65	.04	14	−43	65	0.9
HC vs ARFID–sensory sensitivity group^h^								
ACC	R	4.70	342	<.001	9	8	44	1.18
Sensory association cortex/BA 5	L	4.57	149	.001	−12	−45	62	0.92
	R	4.4	72	.03	15	−46	62	0.87

Abbreviations: ACC, anterior cingulate cortex; ARFID, avoidant/restrictive food intake disorder; BA, Brodmann area; HC, healthy control; L, left; NA, no significant cluster; R, right.

^a^ Coordinates are presented in Montreal Neurological Institute space.

^b^ R/L denotes hemisphere in which peak voxel within each cluster was localized.

^c^ Cluster size (contiguous voxels).

^d^ Statistical significance was assessed at *P* < .05, family-wise error–corrected using cluster-wise correction across the whole brain.

^e^ Data for 35 HC participants and 75 participants with ARFID.

^f^ Data for 35 HC participants and 20 participants with the ARFID-fear phenotype.

^g^ Data for 35 HC participants and 48 participants with the ARFID–lack of interest phenotype.

^h^ Data for 35 HC participants and 58 participants with the ARFID–sensory sensitivity phenotype.

### ARFID-Fear Phenotype vs HC

As predicted, the ARFID-fear group showed greater activation, compared with HC, in the left amygdala while viewing food (vs nonfood) images (mean difference, 0.49 [95% CI, 0.16-0.82]; FWE-corrected *P* = .04 at the ROI level) (Table 2, Figure 1B) with a large effect size (*d* = 0.86). However, within the ARFID-fear group, the PARDI fear of aversive consequences subscale was not significantly associated with amgydala activation. At the whole-brain level, there were no significant differences between the ARFID-fear group and HC group (Table 3).

### ARFID–Lack of Interest Phenotype vs HC

**Figure 2.**
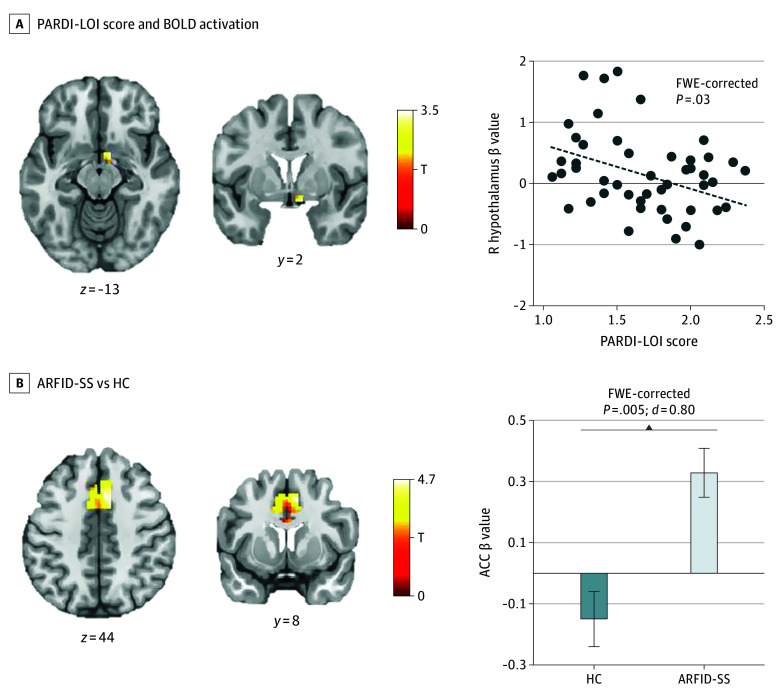
Blood Oxygenation Level–Dependent (BOLD) Activation to Food Cues in Avoidant/Restrictive Food Intake Disorder (ARFID)–Lack of Interest and Sensory Sensitivity Phenotypes Groups (A Priori Region-of-Interest [ROI] Analyses) A, Results showing significant association between Pica, ARFID, and Rumination Disorder Interview lack of interest (PARDI-LOI) score and BOLD activation to foods vs objects in a priori ROIs, within the ARFID–lack of interest phenotype group (48 participants). The T scale and *P* value reflect the bivariate associations from the regression model. Scatterplot (right) depicts values for PARDI-LOI score and mean β values within the cluster. B, Results showing significant differences between groups (healthy control [HC] and ARFID–sensory sensitivity [ARFID-SS]) in BOLD activation to foods vs objects in a priori ROIs. BOLD activation differed between groups (34 HC participants; 58 participants with ARFID-SS) in the anterior cingulate cortex (ACC). The T scale and *P* value reflect the group difference from the independent samples *t* test. Bar graph (right) depicts mean β values within each cluster for each group, with whiskers indicating the SEM. For both panels (A, B), statistical thresholding reflects small volume correction within an anatomically defined bilateral ROI at family-wise error–corrected *P* < .05. Statistical maps for BOLD activation are overlaid on a normalized canonical image (Montreal Neurological Institute ICBM 152 nonlinear asymmetric T1 template) with SPM color map corresponding to relative *t*-value. Coordinates (y and z) are presented in MNI space, with y corresponding to the coronal plane and z to the axial plane.

Contrary to hypotheses, at the ROI level, the ARFID–lack of interest group did not show lower hypothalamus activation vs HC while viewing food (vs nonfood) images. At the whole-brain level, relative to HC, the ARFID–lack of interest group exhibited greater activation in the ACC (mean difference, 0.48 [95% CI, 0.30 to 0.66]; FWE-corrected *P* = .001) and bilateral sensory association cortex (left mean difference, 0.56 [95% CI, 0.30 to 0.82]; FWE-corrected *P* = .006; right mean difference, 0.57 [95% CI, 0.30 to 0.84]; FWE-corrected *P* = .04) (Table 3; eFigure 3 in Supplement 1). As predicted, within the ARFID–lack of interest group, higher scores on PARDI lack of interest were associated with lower right hypothalamus activation at the ROI level (*t* = 3.33; cluster size, 7; FWE-corrected *P* = .03; coordinates: x = 9; y = 2; z = −13) with a medium effect size (*r* = −0.38 [95% CI, −0.69 to −0.11]) (Figure 2A).

### ARFID–Sensory Sensitivity Phenotype vs HC

Consistent with hypotheses, at the ROI level, the ARFID–sensory sensitivity group showed greater activation to food (vs nonfood) images in the ACC compared with HC participants (mean difference, 0.48 [95% CI, 0.22-0.74]; FWE-corrected *P* = .005) (Table 2, Figure 2B) with a large effect size (*d* = 0.80). However, contrary to hypotheses, groups did not differ in insula activation. At the whole-brain level, the ARFID–sensory sensitivity group showed significantly greater activation in the ACC (mean difference, 0.52 [95% CI, 0.33-0.71]; FWE-corrected *P *< .001) and bilateral sensory association cortex (left mean difference, 0.60 [95% CI, 0.33-0.87]; FWE-corrected *P* = .001; right mean difference, 0.54 [95% CI, 0.29-0.80], FWE-corrected *P* = .03) (Table 3; eFigure 4 in Supplement 1). In the regression model, severity of sensory sensitivity was not association with activation in either ACC or insula.

## Discussion

To our knowledge, this is the first functional neuroimaging study to compare children, adolescents, and young adults with ARFID—a relatively new *DSM-5* disorder with unknown neurobiology—to HC participants. Findings suggest that ARFID is characterized by hyperactivation to food stimuli in regions associated with aversive conditioning, attention, and multisensory processing, with variation according to phenotype.

Specifically, results indicate generalized hyperactivation of the ACC, sensory association cortex, and supplementary motor cortex in response to visual food stimuli in ARFID. Hyperactivation in regions associated with attention and cognitive interference (ACC) and multisensory perception and integration (sensory association cortex) appeared to be driven by ARFID–lack of interest and ARFID–sensory sensitivity phenotypes, groups that had high membership overlap in our sample (as is common in clinical practice).^9,10,11^ Importantly, our findings differ from neuroimaging findings in AN. Current results contrast with a previous study from our group that found adolescent females with AN and atypical AN showed greater activation of reward regions (ie, hippocampus, caudate, putamen) in response to high-calorie food images (vs objects), compared with HC participants.^43^ Indeed, prevailing neurobiological models posit that AN is characterized by hypersensitivity of bottom-up reward regions, which requires top-down management of hyperactive cognitive control regions to maintain the restrictive eating driven by desire for thinness that defines the disorder.^43,48,49^ In contrast, we did not observe hyperactivation of reward regions in ARFID in our whole-brain analysis. Instead, ARFID was characterized by hyperactivation in regions associated with cognitive interference, fear conditioning, and multisensory processing, suggesting a distinct neurobiological mechanism for this eating disorder, unassociated with shape and weight concerns.

We also found evidence that neural activation to food cues varies by ARFID phenotype. Specifically, as hypothesized, participants with the ARFID-fear phenotype showed hyperactivation of the amygdala, a key region for aversive conditioning and processing of fearful stimuli, relative to HC participants. This finding is consistent with amygdala hyperactivation reported in individuals with specific phobia^16^ and posttraumatic stress disorder.^17^ Furthermore, although individuals with ARFID–lack of interest did not show between-group differences in hypothalamus hypoactivation compared with HC participants, within the ARFID–lack of interest group itself, hypothalamus activation was inversely correlated with lack of interest severity. Hypoactivation of this key appetite region is consistent with the clinical presentation of ARFID–lack of interest, characterized by low hunger and early satiety. Lastly, contrary to hypotheses related to disgust processing in the ARFID–sensory sensitivity group, these individuals did not differ from HC on insula activation, but exhibited hyperactivation in multisensory integration regions, including the sensory association cortex. Connectivity between the sensory association cortex and motor regions (such as supplementary motor cortex) is elevated during the ingestion of bitter tastants,^50^ and sensory association activation is associated with food preference rating.^51^

### Limitations

Limitations should be noted. First, our sample size was modest for certain phenotype comparisons, resulting in some findings being significant at the ROI level but not at whole-brain level. Second, phenotype overlap prevented us from ruling out whether neural activation was unique to the pure vs combined phenotypes. Third, the use of a passive viewing task required us to reason backward from observed differences in activation between ARFID and HC.^52^ Fourth, psychotropic medication use and a wide age range contributed to sample heterogeneity (however, brain activation to food cues among children parallels that of adults).^53^ Fifth, our sample was predominantly non-Hispanic White and may not generalize to other groups.

## Conclusions

In summary, findings of this fMRI study comparing youths with ARFID with healthy control participants highlight key differences in neural activation in regions associated with aversive conditioning, attention, and multisensory processing. Activation varied by clinical phenotype, which provides insights into neural disruptions to target in future interventional studies.

## Notes

Supplement 1. **eFigure 1.** Flowchart of Participants Included in This Analysis **eFigure 2.** BOLD Activation to Food Cues in ARFID vs Healthy Controls (Whole-Brain Analyses) **eFigure 3.** BOLD Activation to Food Cues in ARFID–Lack Of Interest vs Healthy Controls (Whole-Brain Analyses) **eFigure 4.** BOLD Activation to Food Cues in ARFID–Sensory Sensitivity vs Healthy Controls (Whole-Brain Analyses)

Supplement 2.
Data Sharing Statement
