# Supplementary material for: Neural Response to Food Cues in Avoidant/Restrictive Food Intake Disorder
Source: JAMA Netw Open. 2025 Feb 18;8(2):e2460101. doi: 10.1001/jamanetworkopen.2024.60101 (PMC11836757; doi:10.1001/jamanetworkopen.2024.60101)
Supplement: Supplement 1. — eFigure 1. Flowchart of Participants Included in This Analysis eFigure 2. BOLD Activation to Food Cues in ARFID vs Healthy Controls (Whole-Brain Analyses) eFigure 3. BOLD Activation to Food Cues in ARFID–Lack Of Interest vs Healthy Controls (Whole-Brain Analyses) eFigure 4. BOLD Activation to Food Cues in ARFID–Sensory Sensitivity vs Healthy Controls (Whole-Brain Analyses) [file jamanetwopen-e2460101-s001.pdf]

Supplemental Online Content

Thomas JJ, Holsen L, Van De Water AL, et al. Neural response to food cues in avoidant/restrictive food intake disorder. *JAMA Netw Open.* 2025;8(2):e2460101. doi:10.1001/jamanetworkopen.2024.60101

eFigure 1. Flowchart of Participants Included in This Analysis

eFigure 2. BOLD Activation to Food Cues in ARFID vs Healthy Controls (Whole-Brain Analyses)

eFigure 3. BOLD Activation to Food Cues in ARFID–Lack Of Interest vs Healthy Controls (Whole-Brain Analyses)

eFigure 4. BOLD Activation to Food Cues in ARFID–Sensory Sensitivity vs Healthy Controls (Whole-Brain Analyses)

This supplemental material has been provided by the authors to give readers additional information about their work.

© 2025 Thomas JJ et al. *JAMA Network Open*.

eFigure 1. Flowchart of Participants Included in This Analysis

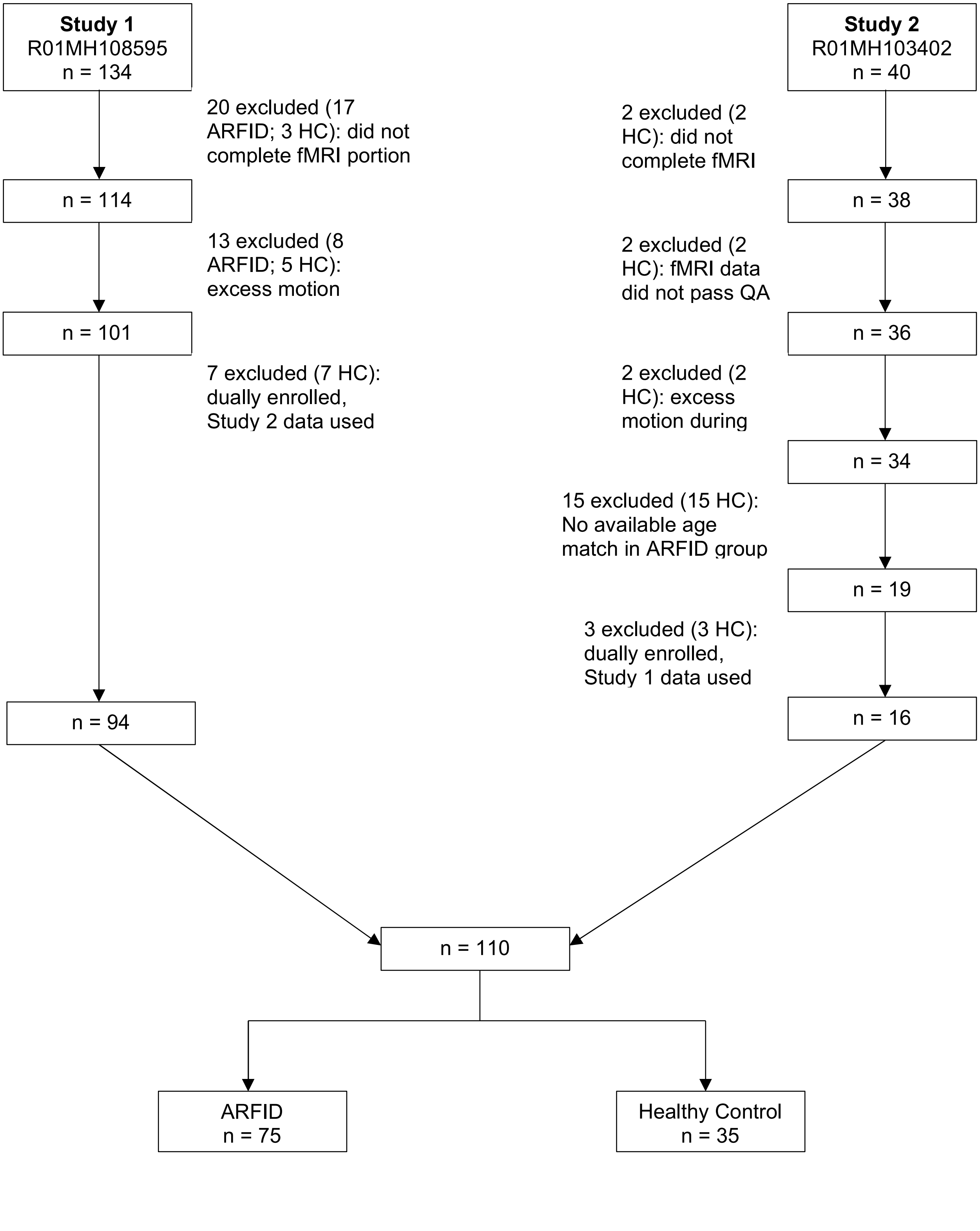

eFigure 1 legend. ARFID=avoidant/restrictive food intake disorder; HC=healthy control.

© 2025 Thomas JJ et al. *JAMA Network Open*.

eFigure 2. BOLD Activation to Food Cues in ARFID vs Healthy Controls (Whole-Brain Analyses)

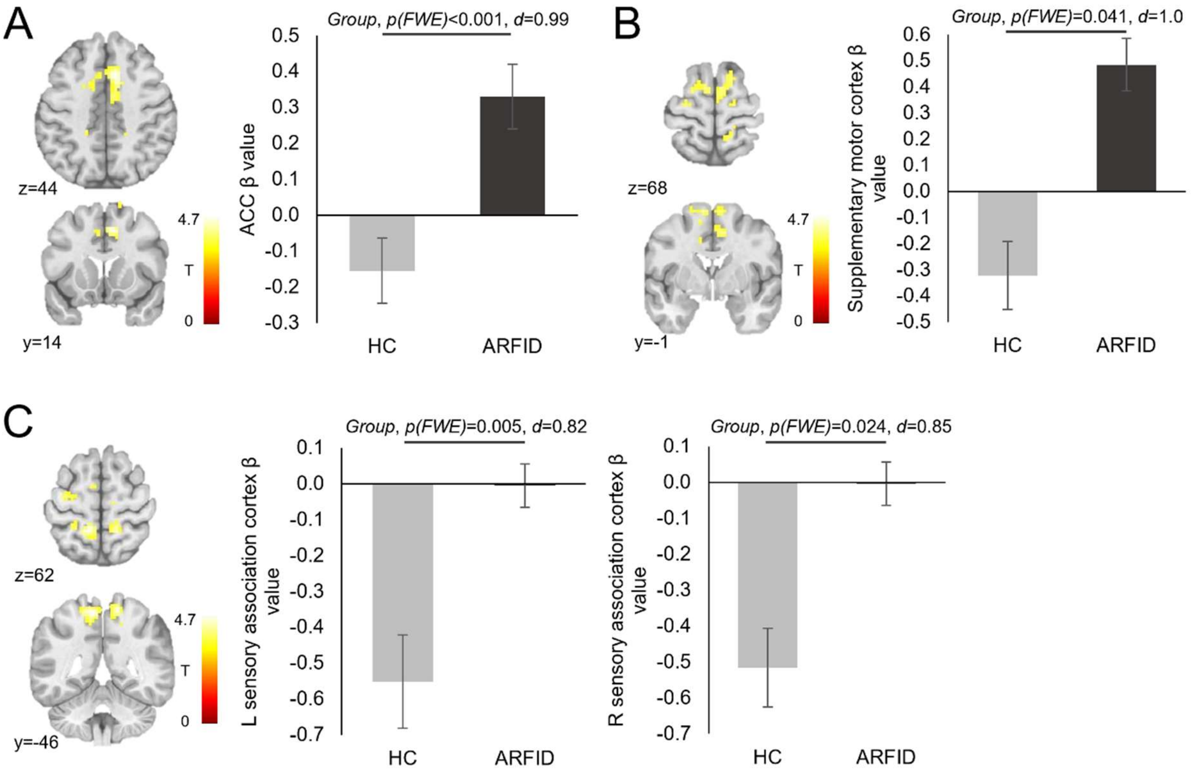

eFigure 2 Legend. Results showing significant differences between groups (healthy control [HC]; ARFID) in blood oxygenation level-dependent (BOLD) activation to foods versus objects at the whole-brain level. BOLD activation differed between groups (HC: *n*=34; ARFID: *n*=75) in the (A) anterior cingulate cortex (ACC), (B) supplementary motor cortex, and (C) bilateral sensory association cortex. The *t* scale and *p* values reflect the group difference from the independent samples t-test. Statistical thresholding reflects whole-brain, cluster level correction at *p*(FWE-corrected)<0.05. Statistical maps for BOLD activation are overlaid on a normalized canonical image (Montreal Neurological Institute [MNI] ICBM 152 nonlinear asymmetric T1 template) with SPM color map corresponding to relative *t*-value. Coordinates (y,z) are presented in MNI space, with y corresponding to the coronal plane and z to the axial plane. Bar graph (right) depicts mean β values within each cluster for each group±*SEM*.

© 2025 Thomas JJ et al. *JAMA Network Open*.

eFigure 3. BOLD Activation to Food Cues in ARFID–Lack Of Interest vs Healthy Controls (Whole-Brain Analyses)

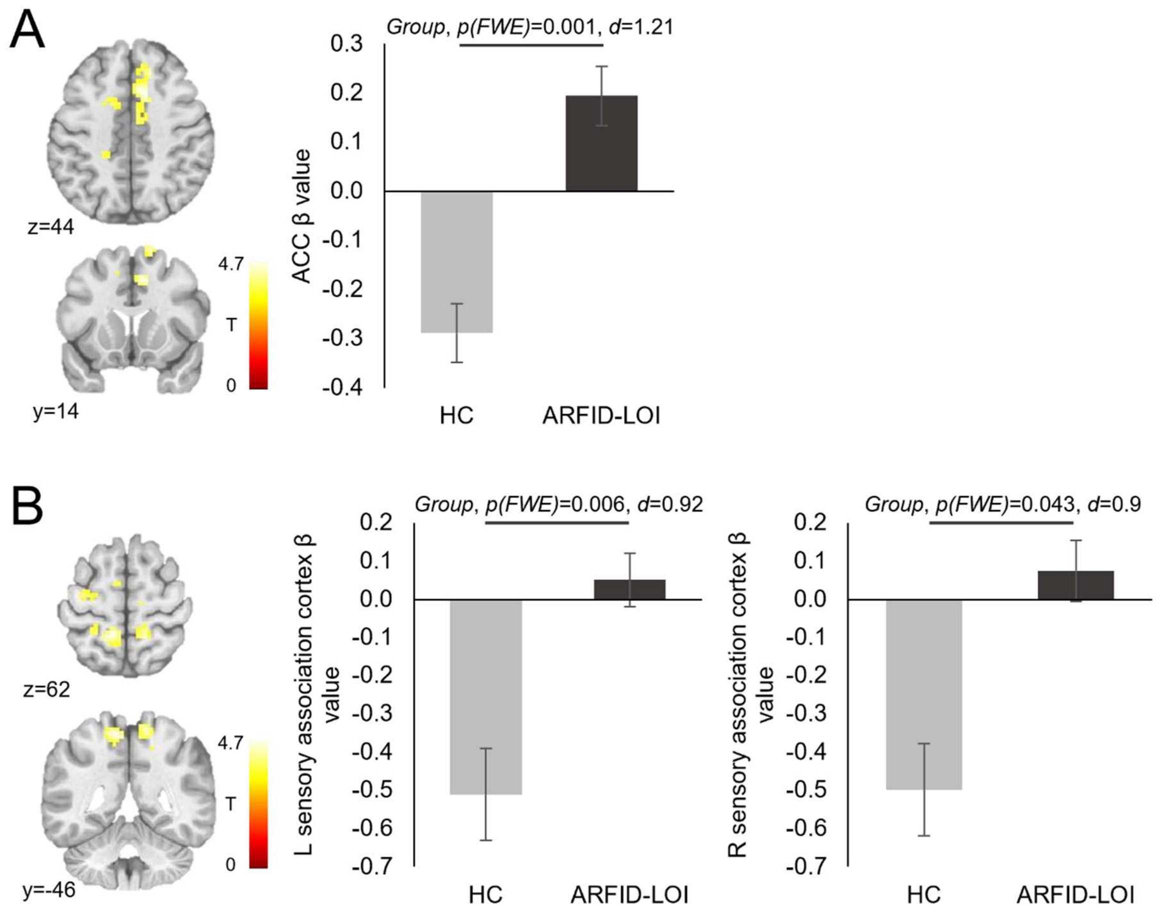

eFigure 3 Legend. Results showing significant differences between groups (healthy control [HC]; *ARFID-lack of interest* [ARFID-LOI]) in blood oxygenation level-dependent (BOLD) activation to foods versus objects at the whole-brain level. BOLD activation differed between groups (HC: *n*=34; ARFID-LOI: *n*=48) in the (A) anterior cingulate cortex (ACC) and (B) bilateral sensory association cortex. The *t* scale and *p* values reflect the group difference from the independent samples t-test. Statistical thresholding reflects whole-brain, cluster level correction at *p*(FWE-corrected)<0.05. Statistical maps for BOLD activation are overlaid on a normalized canonical image (Montreal Neurological Institute [MNI] ICBM 152 nonlinear asymmetric T1 template) with SPM color map corresponding to relative *t*-value. Coordinates (y,z) are presented in MNI space, with y corresponding to the coronal plane and z to the axial plane. Bar graph (right) depicts mean β values within each cluster for each group±*SEM.*

© 2025 Thomas JJ et al. *JAMA Network Open*.

eFigure 4. BOLD Activation to Food Cues in ARFID–Sensory Sensitivity vs Healthy Controls (Whole-Brain Analyses)

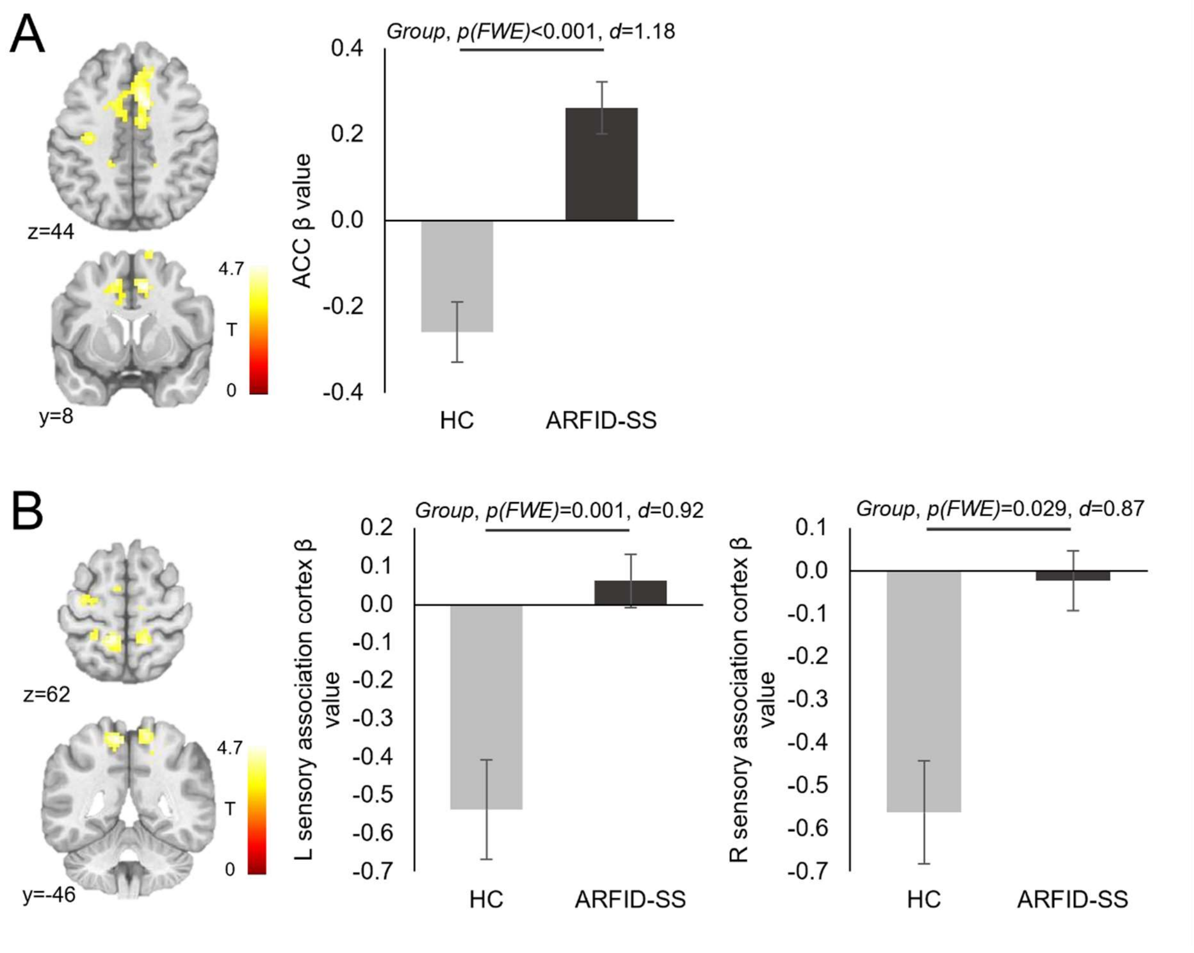

eFigure 4. Results showing significant differences between groups (healthy control [HC]; *ARFID-sensory sensitivity* [ARFID-SS]) in blood oxygenation level-dependent (BOLD) activation to foods versus objects at the whole-brain level. BOLD activation differed between groups (HC: *n*=34; ARFID-SS: *n*=58) in the (A) anterior cingulate cortex (ACC) and (B) bilateral sensory association cortex. The *t* scale and *p* values reflect the group difference from the independent samples t-test. Statistical thresholding reflects whole-brain, cluster level correction at *p*(FWE-corrected)<0.05. Statistical maps for BOLD activation are overlaid on a normalized canonical image (Montreal Neurological Institute [MNI] ICBM 152 nonlinear asymmetric T1 template) with SPM color map corresponding to relative *t*-value. Coordinates (y,z) are presented in MNI space, with y corresponding to the coronal plane and z to the axial plane. Bar graph (right) depicts mean β values within each cluster for each group±*SEM*.

© 2025 Thomas JJ et al. *JAMA Network Open*.
