# Supplementary material for: Neural Response to Food Cues in Avoidant/Restrictive Food Intake Disorder
Source: JAMA Netw Open. 2025 Feb 18;8(2):e2460101. doi: 10.1001/jamanetworkopen.2024.60101 (PMC11836757; doi:10.1001/jamanetworkopen.2024.60101)
Supplement: Supplement 2. — Data Sharing Statement [file jamanetwopen-e2460101-s002.pdf]

Data Sharing Statement

Thomas. Neural Response to Food Cues in Avoidant/Restrictive Food Intake Disorder. *JAMA Netw Open*. Published February 18, 2025. doi:10.1001/jamanetworkopen.2024.60101

Data
Data available: Yes
Data types: Deidentified participant data
How to access data: https://nda.nih.gov
When available: With publication

Supporting Documents
Document types: None

Additional Information
Who can access the data: Anyone requesting the data
Types of analyses: For research purposes
Mechanisms of data availability: Without investigator support, through NIMH National Data Archive
Any additional restrictions: N/A
